# Supplementary material for: A Taybi-Linder syndrome-related RTTN variant impedes neural rosette formation in human cortical organoids
Source: PLoS Genet. 2024 Dec 16;20(12):e1011517. doi: 10.1371/journal.pgen.1011517 (PMC11684760; doi:10.1371/journal.pgen.1011517)
Supplement: S1 Methods — (PDF) [file pgen.1011517.s001.pdf]

# S1 Methods

## Genetic studies

Informed consent for genetic analysis was obtained from the couple. DNA was extracted from blood samples. Trio Exome Sequencing was performed and analysed at the University Hospital of Lyon. Library preparation was performed with the Medexome kit (Roche) following manufacturer's instructions, and paired-end 2x150 sequencing on a NextSeq500 instrument (Illumina). Genomic alignment against the hg19/GRCh37 assembly and variant calling were, respectively, done with BWA-MEM v.0.7.12 (1) and GATK HaplotypeCaller v.3.4 (Broad Institute, Boston, MA, USA) while QC were evaluated using DeCovA (2). Only highly confident variants were kept for analysis (total depth >9; alternative allele depth >4; no strand bias; mosaicism >10%). Rare variants were defined by a minor allele frequency below 1% in gnomAD database. Data are available on request from the authors. A Sanger validation was performed to confirm the candidate variant and parental segregation (S2 Table).

## RNA extraction, RT-PCR and RT-qPCR

RNA was either extracted with the Nucleospin Tissue kit (Macherey-Nagel, 740952.50) following manufacturer's instructions, or with chloroform-trizol standard protocol, and 1.5 µg of RNA was treated with 1 U/µg of DNase I (ThermoFisher, EN0521) for 10 minutes at 37°C. To stop the reaction, 1x of EDTA (ThermoFisher, EN0521) was added and incubated for 10 minutes at 60°C. Then, 1 µg of DNA-clear RNA was reverse transcribed using the GoScript kit (Promega, A5001) following manufacturer's instructions, with a mixture of oligodT and random primers. For PCR, 100 ng of cDNA was mixed with 1X GoTaq green master mix (Promega, M7122) and 0.5 µM primer mix (forward and reverse, S2 Table) in a total volume of 20 µL. For RT-qPCR, 6.25 or 25 ng of cDNA was used per reaction with 0.5 µM of primer mix (S2 Table) and 1X ONEgreen fast qPCR premix (Ozyme, OZYA008) in a total volume of 20 µL, using the Rotor-gene Q (Qiagen).

## Generation of RPE1-based doxycycline-inducible cell lines

To generate doxycycline-inducible expression of mutated RTTN-GFP in *RTTN*-dKO RPE1 cells, we used a previously reported construct consisting of the GFP-tagged *RTTN* cDNA cloned into pLVX-Tight-Puro vector (3). The missense variant p.Arg985Gly (leading to a protein isoform named RG) was generated by site-directed mutagenesis using the QuikChange kit, and the exon 23 deletion, causing the loss of amino-acids 963-985 (Δ23 protein isoform), was obtained by RT-PCR amplification from pFlag-tagged WT RTTN construct and sub-cloned into in-frame pLVX-Tight-Puro vector. Both constructs were confirmed by Sanger sequencing. Then, lentiviruses were produced as previously described (3), and used to infect *RTTN*-dKO RPE1 Tet-On cells stably expressing rtTA. The infected cells were selected with puromycin (10 μg/ml) for 7 days or by cell sorter on the basis of GFP fluorescence and expanded. The expression of mutated RTTN-GFP (RG or Δ23) was induced by doxycycline (1 μg/ml) treatment as previously described (3) and verified by immunofluorescence and western blot (S3A and S3B Fig).

## CRISPR/Cas9-mediated genome editing in iPS cells

For CRISPR/Cas9-mediated insertion of *RTTN* c.2953G>A variant, cr-RNAs were designed using CRISPOR-tefor (http://crispor.tefor.net/) tool (4). The guide RNA assembly was prepared as followed: 400 µM of each of the tracr-RNA (IDT, 1075928) and cr-RNA (IDT, S2 Table) were mixed and incubated in a thermocycler using the following program: 94°C for 4 min, 75 cycles of 94°C 5 sec + 93,5°C 5 sec with each cycle an increment of 1°C, 20°C for 7 min. For transfection using the Nucleofector 4D (Lonza), 400,000 single CAU2 iPS cells, previously incubated for 1h with 10 µM Y-27632, were mixed with the P3 primary cell solution and Supplement 1 (both from Lonza, V4XP-3032), 62 pmol HiFi Caspase9 (IDT, 1081060), 400 pmol guide RNA and 300 pmol ssODN (IDT, S2 Table). This ssODN matrix contains, in addition to the c.2953G>A variant, a nucleotide change at the PAM sequence, predicted to not alter *RTTN* expression nor splicing, which avoids further cutting from the Cas9. After the electric shock, cells were allowed to recover in hot mTesR Plus

medium supplemented with 10 µM Y-27-632 for 10 minutes, before being transferred into laminin 521-coated wells (STEMCELL Technologies, 77004). Medium was changed every other day, without adding Y-27632, until cells reached confluence and transferred onto vitronectin-coated dish at very low density (12.5 or 25 cells/cm²) in mTesR Plus medium supplemented with 10% CloneR (STEMCELL Technologies, 05888). When colonies were large enough, each colony was scrapped to perform Sanger sequencing (S5A Fig). Sequencing showed that 20% of all screened iPSC colonies presented a bi-allelic knock-in (KI) of the variant. Four clones with normal morphology and the desired genotypes (two WT and two KI) were controlled for genomic integrity using iCSdigital probes from Stem Genomics (S5B Fig), sequencing of predicted off-targets by CRISPOR-tefor (4) (S5C Fig), and G-banding karyotyping (S5D Fig). All correctly expressed *RTTN* with the presence of the three different isoforms in both KI clones (S5E-S5G Fig).

## Ultrastructural expansion microscopy (U-ExM)

The U-ExM protocol was performed as previously reported (5, 6). Briefly, cells were seeded at a density of 75,000 cells/cm² on coverslips and the following day, were incubated with molecular anchors (1.4% formaldehyde (Sigma, F8776) and 2% acrylamide (Sigma, A4058)) for 3h at 37°C. Coverslips were then placed on top of 38 µL of ice-cold monomeric solution (1X PBS, 23% sodium acrylate (AK Scientific, R426), 10% acrylamide, 0.1% N,N′-methylenbisacrylamide (Sigma, M1533)) with 5% TEMED (ThermoFisher, 17919) and 5% ammonium persulfate (APS, ThermoFisher, 17874) placed on Parafilm, and incubated for 1h at 37°C for polymerization to happen, followed by a denaturation at 95°C for 1h30. The following day, a quarter of gel was incubated with primary antibodies diluted in 1X PBS, 2% BSA and incubated for 3h at 37°C with orbital agitation. After three washes with 1X PBS + 0.1% Tween20, secondary antibodies were incubated as previously done for primary antibodies but in the dark. After three more washes, the gel was expanded in water overnight. Upon imaging, a small piece of gel was cut and put on top of a poly-D-lysine (0.1 mg/ml,

A3890401, Gibco)-coated coverslip. The Zeiss LSM 800 confocal microscope was used to image the NSC while an inverted Leica Thunder microscope was used to image the fibroblasts.

## References


1. Li H, Durbin R. Fast and accurate short read alignment with Burrows-Wheeler transform. Bioinformatics. 2009;25(14):1754-60.

2. Dimassi S, Simonet T, Labalme A, Boutry-Kryza N, Campan-Fournier A, Lamy R, et al. Comparison of two next-generation sequencing kits for diagnosis of epileptic disorders with a user-friendly tool for displaying gene coverage, DeCovA. Appl Transl Genom. 2015;7:19-25.

3. Chen HY, Wu CT, Tang CC, Lin YN, Wang WJ, Tang TK. Human microcephaly protein RTTN interacts with STIL and is required to build full-length centrioles. Nature communications. 2017;8(1):247.

4. Concordet JP, Haeussler M. CRISPOR: intuitive guide selection for CRISPR/Cas9 genome editing experiments and screens. Nucleic acids research. 2018;46(W1):W242-W5.

5. Gambarotto D, Hamel V, Guichard P. Ultrastructure expansion microscopy (U-ExM). Methods in cell biology. 2021;161:57-81.

6. Khatri D, Putoux A, Cologne A, Kaltenbach S, Besson A, Bertiaux E, et al. Deficiency of the minor spliceosome component U4atac snRNA secondarily results in ciliary defects in human and zebrafish. Proceedings of the National Academy of Sciences of the United States of America. 2023;120(9):e2102569120.
