# Supplementary material for: A Taybi-Linder syndrome-related RTTN variant impedes neural rosette formation in human cortical organoids
Source: PLoS Genet. 2024 Dec 16;20(12):e1011517. doi: 10.1371/journal.pgen.1011517 (PMC11684760; doi:10.1371/journal.pgen.1011517)
Supplement: S1 Fig — (PDF) [file pgen.1011517.s002.pdf]

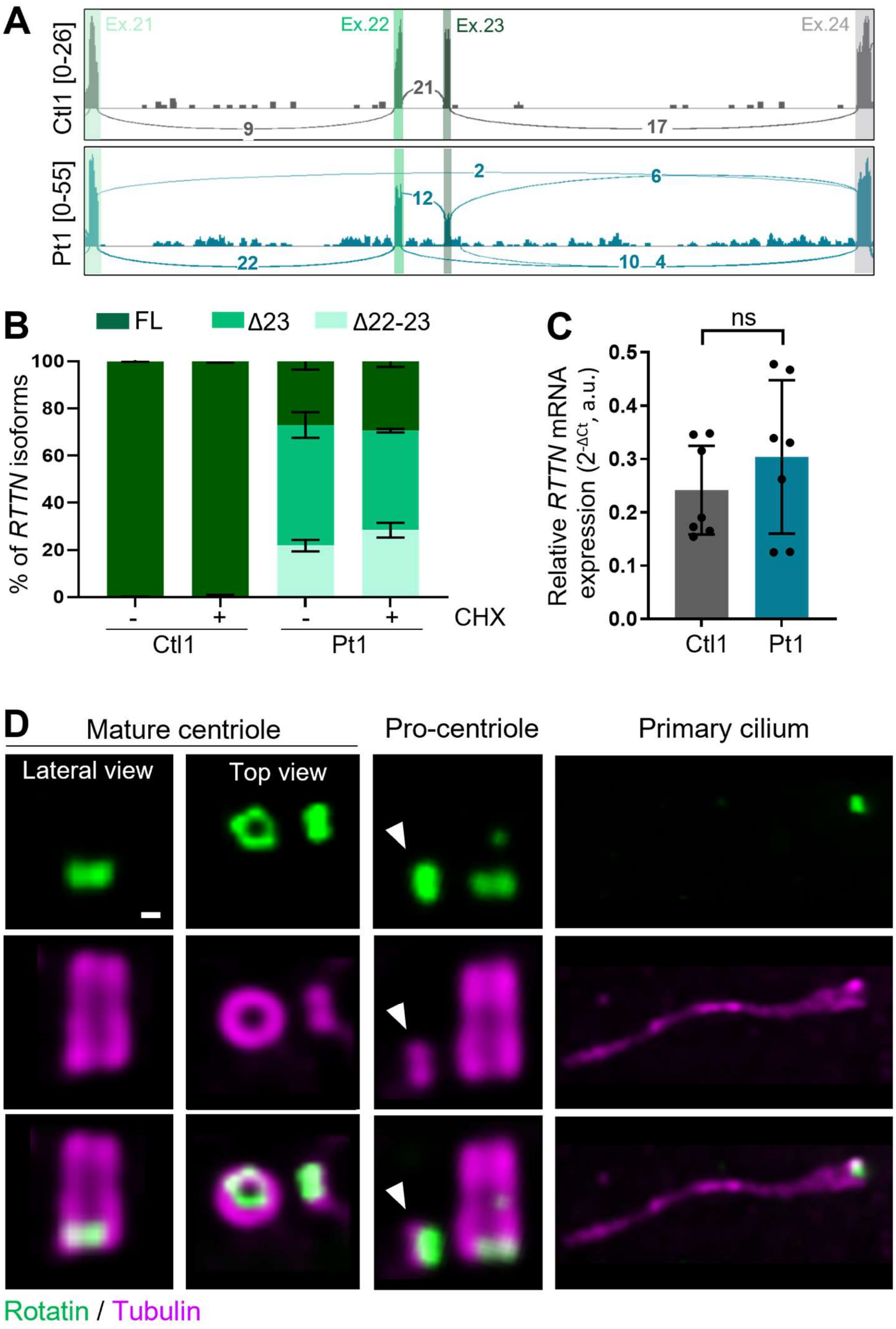

S1 Fig. Characterization of the impact of *RTTN* c.2953A>G variant on *RTTN* pre-mRNA splicing and global expression in patient fibroblasts. All experiments were performed in control (Ctl1) and patient (Pt1) fibroblasts. **(A)** Sashimi plot visualization of aligned RNA sequencing data from fibroblasts at *RTTN* introns 21 to 23. On the arcs, the number of junction-spanning reads supporting exon pairs; in brackets, the range of coverage of each base of the depicted region**. (B, C)** RT-qPCR analyses of each of the *RTTN* splicing isoforms (B), in absence (-) or presence (+) of the NMD inhibitor cycloheximide (CHX), and of the relative global expression (C) in fibroblasts. *RPS17*

was used as the house-keeping gene. Graph (C) shows the mean ± SD of seven independent experiments. Differences are not significant (ns) by Mann-Whitney's test. **(D)** Representative confocal images of Rotatin (green) localisation at expanded centrioles (lateral or top views) and at the base of primary cilium (α/β-Tubulin, magenta) in control fibroblasts. Scale bar: 100 nm. a.u., arbitrary units.
