# Supplementary material for: A Taybi-Linder syndrome-related RTTN variant impedes neural rosette formation in human cortical organoids
Source: PLoS Genet. 2024 Dec 16;20(12):e1011517. doi: 10.1371/journal.pgen.1011517 (PMC11684760; doi:10.1371/journal.pgen.1011517)
Supplement: S2 Fig — (PDF) [file pgen.1011517.s003.pdf]

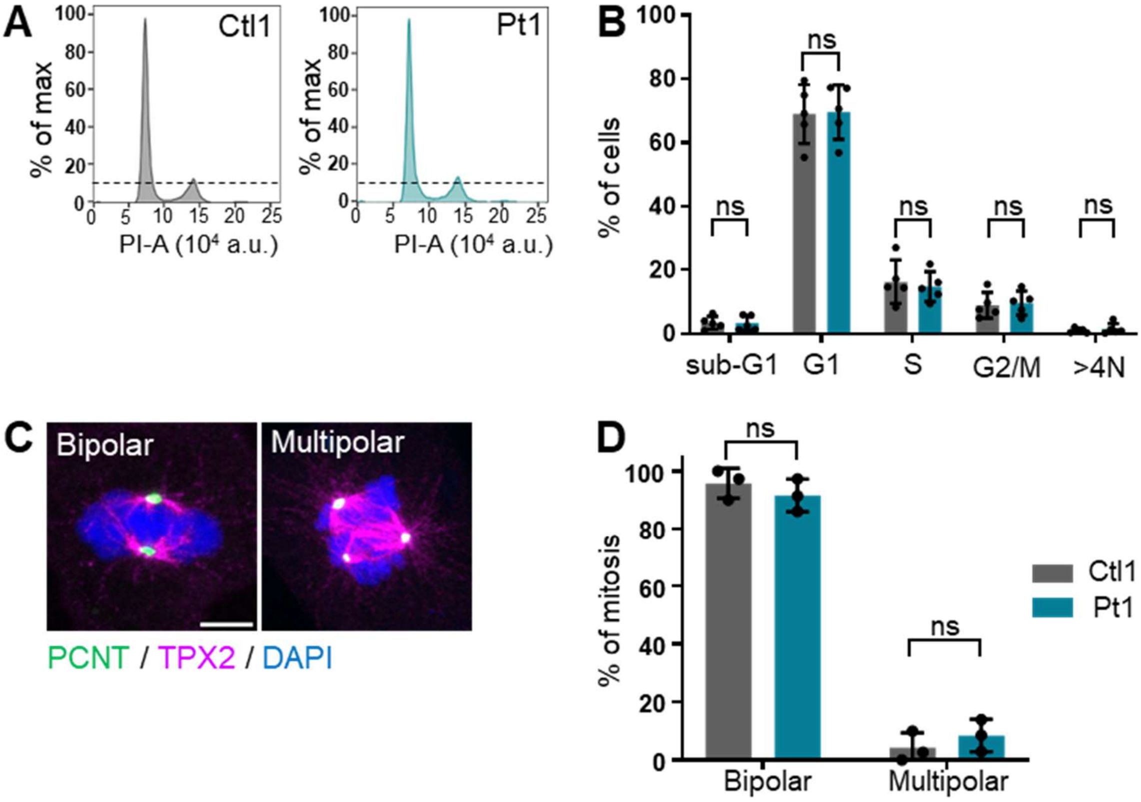

S2 Fig. Characterization of the impact of *RTTN* c.2953A>G variant on cell cycle progression and mitosis in patient fibroblasts. All experiments were performed in control (Ctl1) and patient (Pt1) fibroblasts. **(A)** Flow cytometric cell cycle analysis histograms in fibroblasts. The dotted line represents the top of G2/M peak in Ctl1 cells for reference. **(B)** Quantification of the percentage of cells in each cell cycle phase. Graph shows the mean ± SD of five independent experiments. **(C)** Confocal images of representative normal (bipolar) and abnormal (multipolar) mitosis events seen in fibroblasts. Pericentrin (PCNT) stains centrosomes, TPX2 mitotic spindles and DAPI DNA. **(D)** Quantification of the proportion of normal and abnormal mitosis events such as seen in C. Graph shows the mean ± SD of three independent experiments (n>100 mitosis per experiment). Differences are not significant (ns) by two-way ANOVA with Tukey's correction (B, D). Scale bar: 5 µm. a.u., arbitrary units; PI, propidium iodide.
