# Supplementary material for: A Taybi-Linder syndrome-related RTTN variant impedes neural rosette formation in human cortical organoids
Source: PLoS Genet. 2024 Dec 16;20(12):e1011517. doi: 10.1371/journal.pgen.1011517 (PMC11684760; doi:10.1371/journal.pgen.1011517)
Supplement: S3 Fig — (PDF) [file pgen.1011517.s004.pdf]

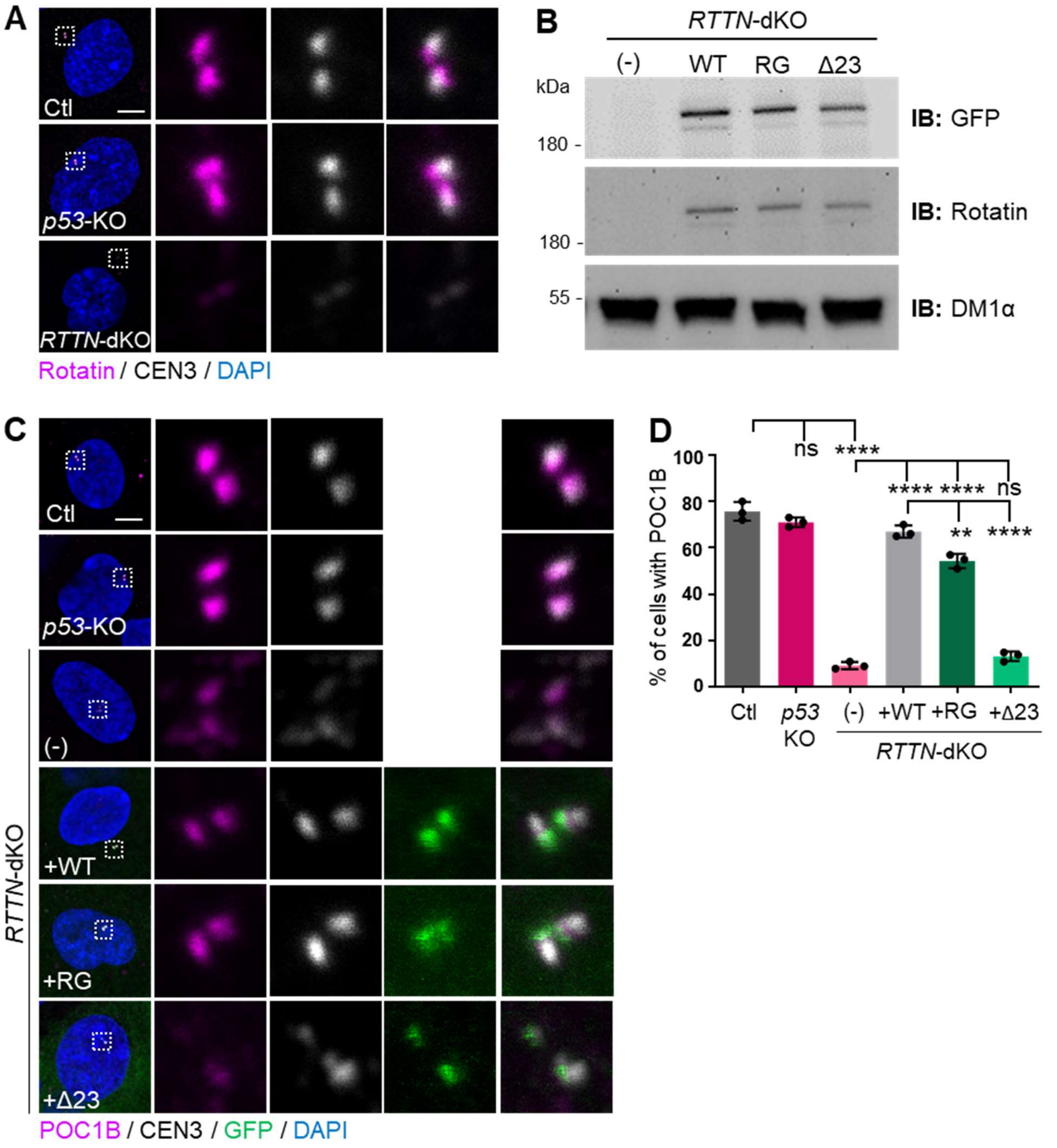

S3 Fig. Characterization of *RTTN*-dKO RPE1 cellular model and analysis of POC1B centriolar localisation. All experiments were performed in control, *p53*-KO and *RTTN*-dKO RPE1 cells induced to express wild-type (WT) or mutated (RG, Δ23) RTTN-GFP proteins. **(A)** Representative confocal images of Rotatin protein expression (magenta) in *RTTN*-dKO RPE1 cellular model. CEN3 labels centrioles. **(B)** Western blot analysis of Rotatin protein expression, with the indicated antbodies, in *RTTN*-dKO RPE1 cellular model. α-Tubulin (DM1α) is used as a loading control. **(C)** Confocal images of POC1B (magenta) localization at centrosomes (CEN3, grey) in *RTTN*-dKO RPE1

cellular model. **(D)** Quantitative analysis of cells expressing POC1B protein at the centrioles such as seen in C. Graph shows the mean ± SD from three independent experiments (100 cells per experiment). ns, not significant; **p-value< 0.01; ****p-value<0.0001 following one-way ANOVA with Tukey's multiple comparison test. Scale bar: 5 μm. DAPI stains DNA.
