# Supplementary material for: A Taybi-Linder syndrome-related RTTN variant impedes neural rosette formation in human cortical organoids
Source: PLoS Genet. 2024 Dec 16;20(12):e1011517. doi: 10.1371/journal.pgen.1011517 (PMC11684760; doi:10.1371/journal.pgen.1011517)
Supplement: S4 Fig — (PDF) [file pgen.1011517.s005.pdf]

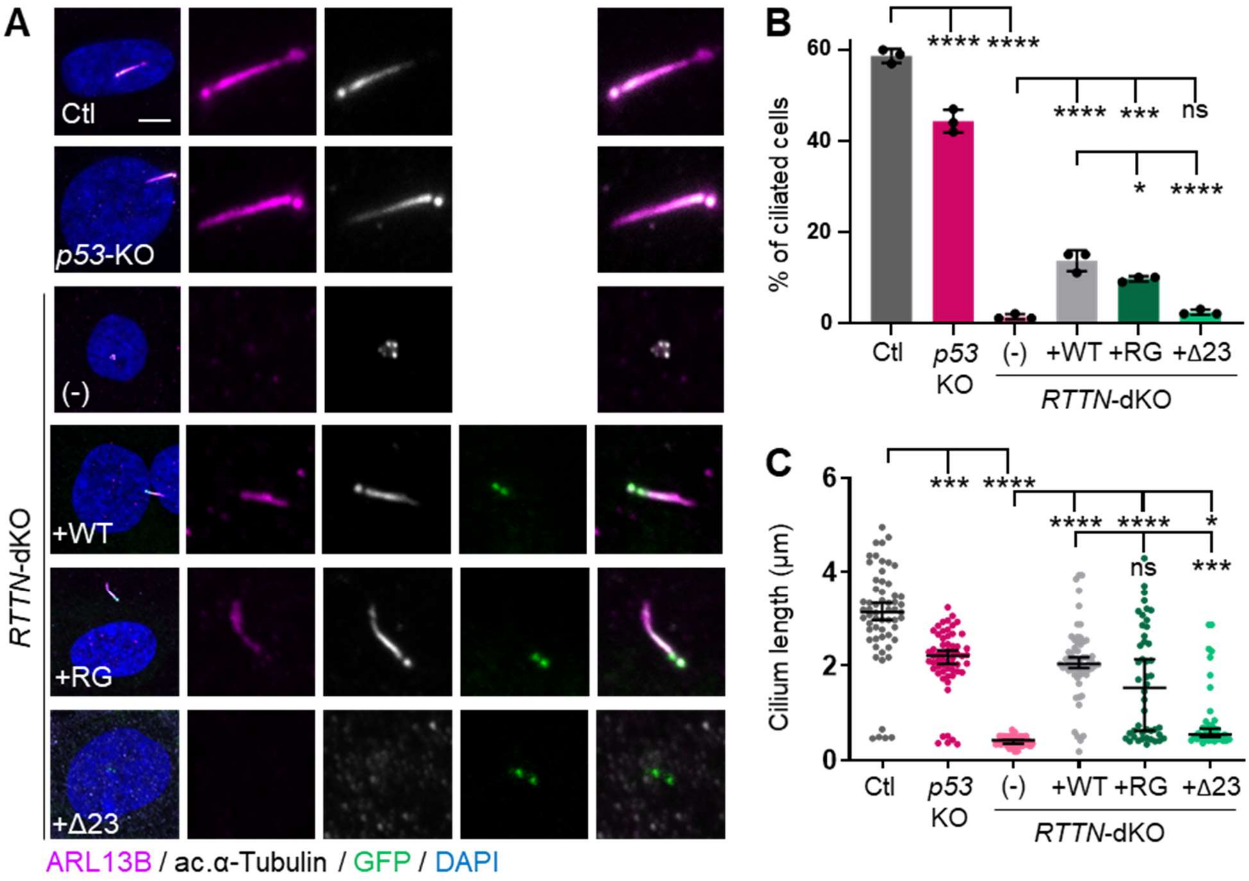

S4 Fig. Characterization of the impact of p.Arg985Gly (RG) and Δ23 variants on primary cilium formation in *RTTN*-dKO RPE1 cellular model. All experiments were performed in control, *p53*-KO and *RTTN*-dKO RPE1 cells induced to express wild-type (WT) or mutated (RG, Δ23) RTTN-GFP proteins. **(A)** Representative confocal images of primary cilium, stained with the ciliary membrane marker ARL13B (magenta) and the axonemal marker acetylated-αTubulin (grey), in *RTTN*-dKO RPE1 cellular model. DAPI (blue) labels DNA. **(B-C)** Quantification of the percentage of ciliated cells (B) and length of primary cilium (C) such as seen in A. Graphs show the mean ± SD (B) or the median ± 95% CI (C) of three independent experiments (n>40 cells). ns, not significant; *p-value<0.05; ***p-value<0.005; ****p-value<0.0001 following one-way ANOVA with Dunnett's multiple comparison test (B) or Kruskal-Wallis with Dunn's multiple comparison test (C). Scale bar: 5 μm.
