# Supplementary material for: A Taybi-Linder syndrome-related RTTN variant impedes neural rosette formation in human cortical organoids
Source: PLoS Genet. 2024 Dec 16;20(12):e1011517. doi: 10.1371/journal.pgen.1011517 (PMC11684760; doi:10.1371/journal.pgen.1011517)
Supplement: S5 Fig — (PDF) [file pgen.1011517.s006.pdf]

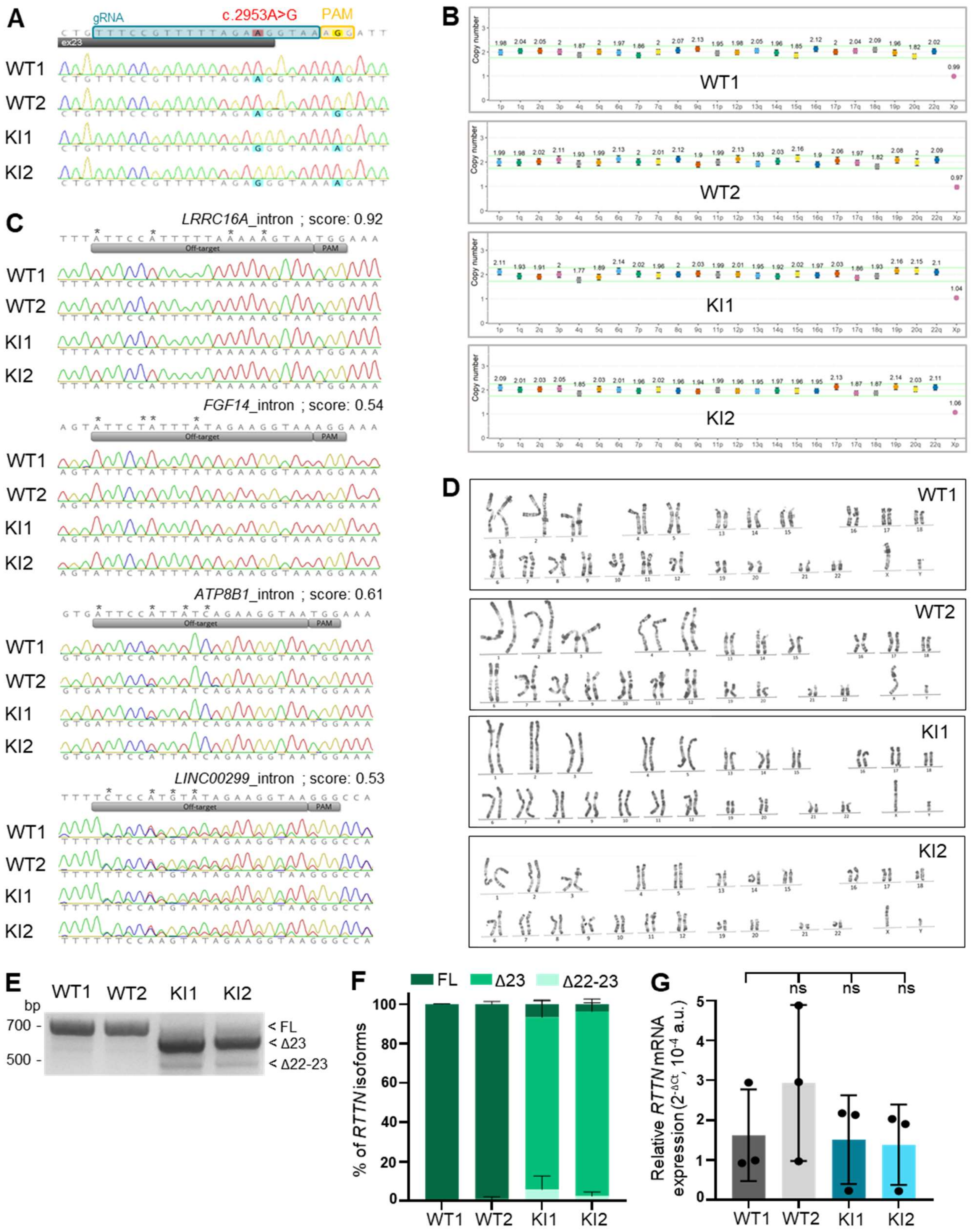

S5 Fig. Validation of the CRISPR/Cas9-mediated genetic modification of *RTTN* gene in four iPS clones. All experiments were performed in two wild-type (WT) and two knock-in (KI) iPS clones. **(A)** Chromatograms of Sanger sequencing of iPS clones showing the region encompassing the *RTTN*

c.2953A nucleotide. Positions of the guide RNA and the PAM sequences are highlighted in blue and yellow, respectively. **(B)** Analysis of the copy number of the most frequent chromosomic variations seen in iPS clones, using iCS-digital PCS test. Each marker is seen in two copies, except the marker on chr. X (male genotype), validating the genomic integrity of the iPS clones. **(C)** Chromatograms of Sanger sequencing of the off-targets predicted with the highest score (>0.4). Asterisks highlight the nucleotide mismatches between the *RTTN* targeted sequence and the off-target. No mutation in the introns of the tested genes was detected. **(D)** G-banding karyotypes of the selected iPS clones. No chromosomic alteration is observed. **(E-F)** RT-PCR (E) and RT-qPCR (F) analyses of the splicing events of *RTTN* exon 23 in iPS clones. **(G)** RT-qPCR analysis of *RTTN* relative expression in iPSC clones normalised to *RPS17*. Graphs (F, G) show the mean ± SD of three independent experiments. Differences are not significant by Kruskal-Wallis test with Dunn's multiple comparisons test. a.u. arbitrary units.
