# Supplementary material for: A Taybi-Linder syndrome-related RTTN variant impedes neural rosette formation in human cortical organoids
Source: PLoS Genet. 2024 Dec 16;20(12):e1011517. doi: 10.1371/journal.pgen.1011517 (PMC11684760; doi:10.1371/journal.pgen.1011517)
Supplement: S6 Fig — (PDF) [file pgen.1011517.s007.pdf]

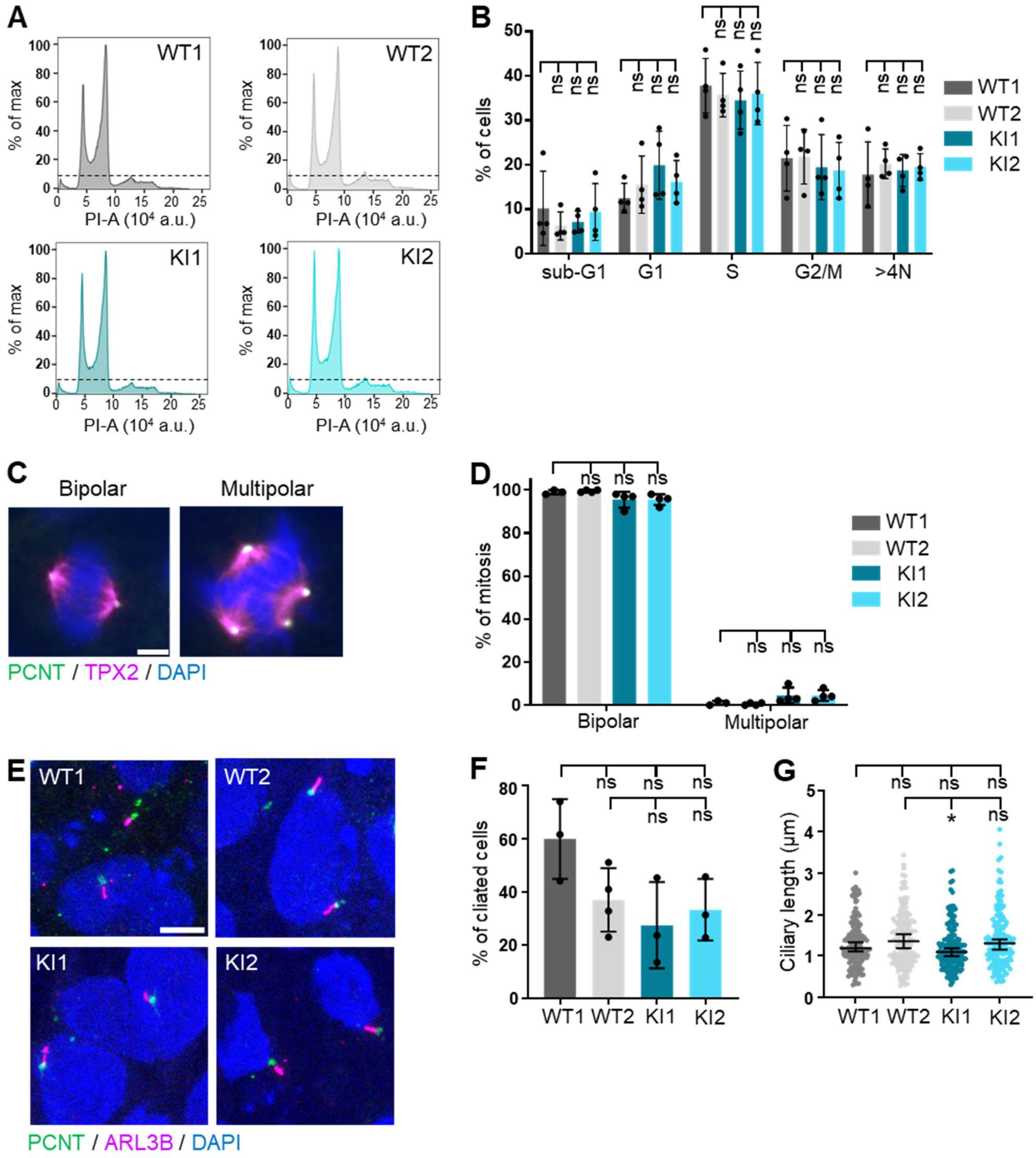

S6 Fig. Characterization of *RTTN*-mutated iPS clone phenotypes. All experiments were performed in two wild-type (WT) and two knock-in (KI) iPS clones. **(A)** Flow cytometric cell cycle analysis histograms in iPS clones. **(B)** Quantification of the percentage of cells in each cell cycle phase. Graph shows the mean ± SD of four independent experiments. **(C)** Confocal images of representative normal (bipolar) and abnormal (multipolar) mitosis events seen in iPS clones. Pericentrin (PCNT) stains centrosomes, TPX2 mitotic spindles. **(D)** Quantification of the proportion

of normal and abnormal mitosis events such as seen in C. Graph shows the mean ± SD of four independent experiments (n>100 mitosis per experiment). **(E)** Representative confocal images of primary cilium (ARL13B, magenta) and centrosome (PCNT, green) in iPS clones. **(F, G)** Quantification of the percentage of ciliated cells (F) and of length of primary cilium (G) such as observed in E. Graphs show the mean ± SD (F) or median ± 95% CI (G) of four independent experiments (n=150 cells per experiment). ns, non-significant; *p-value<0.5 by two-way (B, D) or one-way (F) ANOVA with Tukey's correction, or Kruskal-Wallis test with Dunn's multiple comparisons test (G). Scale bars: 2 µm (B), 5 µm (C). DAPI labels DNA. a.u. arbitrary units.
