# Supplementary material for: A Taybi-Linder syndrome-related RTTN variant impedes neural rosette formation in human cortical organoids
Source: PLoS Genet. 2024 Dec 16;20(12):e1011517. doi: 10.1371/journal.pgen.1011517 (PMC11684760; doi:10.1371/journal.pgen.1011517)
Supplement: S7 Fig — (PDF) [file pgen.1011517.s008.pdf]

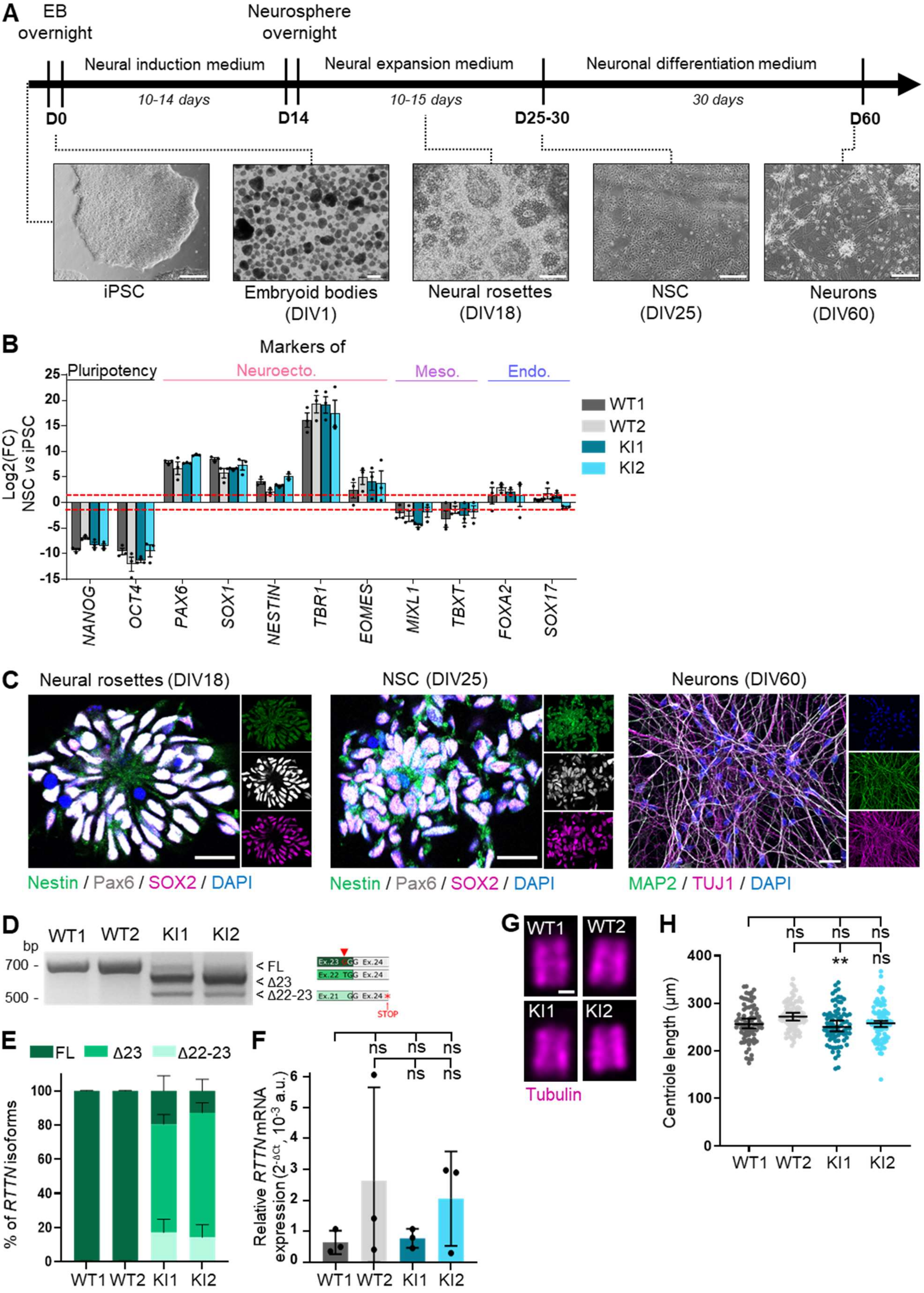
A
EB overnight
Neurosphere overnight
Neural induction medium
Neural expansion medium
Neuronal differentiation medium
10-14 days
10-15 days
30 days
D0
D14
D25-30
D60
iPSC
Embryoid bodies (DIV1)
Neural rosettes (DIV18)
NSC (DIV25)
Neurons (DIV60)
B
Markers of
Pluripotency
Neuroecto.
Meso.
Endo.
Log2(FC) NSC vs iPSC
WT1
WT2
KI1
KI2
NANOG
OCT4
PAX6
SOX1
NESTIN
TBR1
EOMES
MIXL1
TBXT
FOXA2
SOX17
C
Neural rosettes (DIV18)
NSC (DIV25)
Neurons (DIV60)
Nestin / Pax6 / SOX2 / DAPI
Nestin / Pax6 / SOX2 / DAPI
MAP2 / TUJ1 / DAPI
D
WT1 WT2 KI1 KI2
bp
700 -
500 -
< FL
< Δ23
< Δ22-23
STOP
E
FL Δ23 Δ22-23
% of RTTN isoforms
WT1 WT2 KI1 KI2
F
Relative RTTN mRNA expression (2^-ΔCt, 10^-3 a.u.)
ns ns ns
ns ns
WT1 WT2 KI1 KI2
G
WT1 WT2
KI1 KI2
Tubulin
H
ns ns ns
**
ns
Centriole length (µm)
WT1 WT2 KI1 KI2

S7 Fig. 2D differentiation of iPSC into neural stem cells (NSC) and neurons. All experiments were performed in control (WT) and *RTTN*-mutated (KI) NSC harvested at DIV25. **(A)** Timeline of iPSC differentiation into neural stem cells and neurons. Bright field images of cells at the key stages of the differentiation are shown. **(B)** RT-qPCR analysis of gene expression of markers of pluripotency, neuroectoderm, mesoderm and endoderm in NSC compared to iPSC. iPSC express markers of pluripotency while NSC predominantly express markers of neuroectoderm. Graph shows the mean ± SD of three independent experiments. **(C)** Representative confocal images of neural rosettes (left), NSC (middle) and neurons (right). Neural rosettes and NSC are stained for nestin (green), Pax6 (grey) and SOX2 (magenta) while neurons are labelled with MAP2 (green) and TUJ1 (magenta). DAPI stains nuclei. **(D-F)** RT-PCR (D) and RT-qPCR (E) analyses of the splicing events of *RTTN* exon 23, and of the *RTTN* relative expression in NSC. *RPS17* was used as a house-keeping gene. Graphs show the mean ± SD of three independent experiments. **(G)** Representative confocal images of expanded centrioles (Tubulin, magenta) in NSC. **(H)** Quantification of length of centriole such as seen in G. Graph shows the median ± 95% CI of three independent experiments. ns not significant; **p-value<0.01 following Kruskal-Wallis test with Dunn's multiple comparisons test (F) or one-way ANOVA with Tukey's correction (H). Scale bars: 200 µm (A), 20 µm (C), 100 nm (G). a.u. arbitrary units; EB, embryoid bodies.
