# Supplementary material for: A Taybi-Linder syndrome-related RTTN variant impedes neural rosette formation in human cortical organoids
Source: PLoS Genet. 2024 Dec 16;20(12):e1011517. doi: 10.1371/journal.pgen.1011517 (PMC11684760; doi:10.1371/journal.pgen.1011517)
Supplement: S8 Fig — (PDF) [file pgen.1011517.s009.pdf]

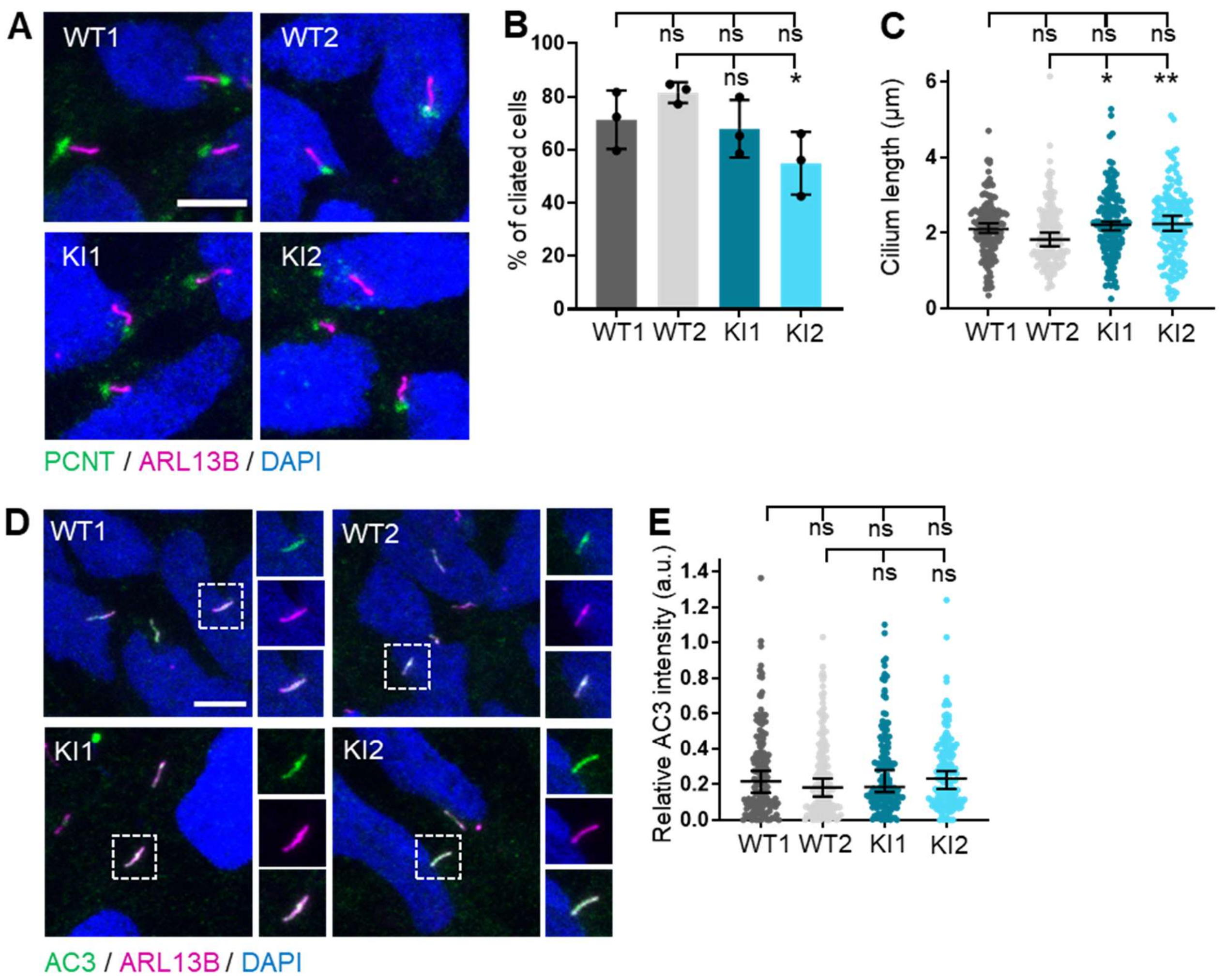

S8 Fig. Characterization of primary cilium formation and function in *RTTN*-mutated NSC. All experiments were performed in control (WT) and *RTTN*-mutated (KI) NSC. **(A)** Representative confocal images of primary cilium (ARL13B, magenta) and centrosome (PCNT, green) in NSC. **(B, C)** Quantification of percentage of ciliated cells (B) and of length of primary cilium (C) such as seen in A. Graphs show the mean ± SD (B) or median ± 95% CI (C) of three independent experiments (n=150 cells). **(D)** Representative confocal images of adenylate cyclase 3 (AC3, green) in primary cilium (ARL13B, magenta) in NSC. **(E)** Quantification of relative AC3 intensity compared to ARL13B. Graph shows the median ± 95% CI of three independent experiments (n=150 cells). ns not significant; *p-value<0.05; **p-value<0.01 following one-way ANOVA with Tukey's correction (B) or Kruskal-Wallis with Dunn's correction (C, E). Scale bars: 5µm. DAPI labels the nuclei. a.u., arbitrary units.
