# Supplementary material for: A Taybi-Linder syndrome-related RTTN variant impedes neural rosette formation in human cortical organoids
Source: PLoS Genet. 2024 Dec 16;20(12):e1011517. doi: 10.1371/journal.pgen.1011517 (PMC11684760; doi:10.1371/journal.pgen.1011517)
Supplement: S9 Fig — (PDF) [file pgen.1011517.s010.pdf]

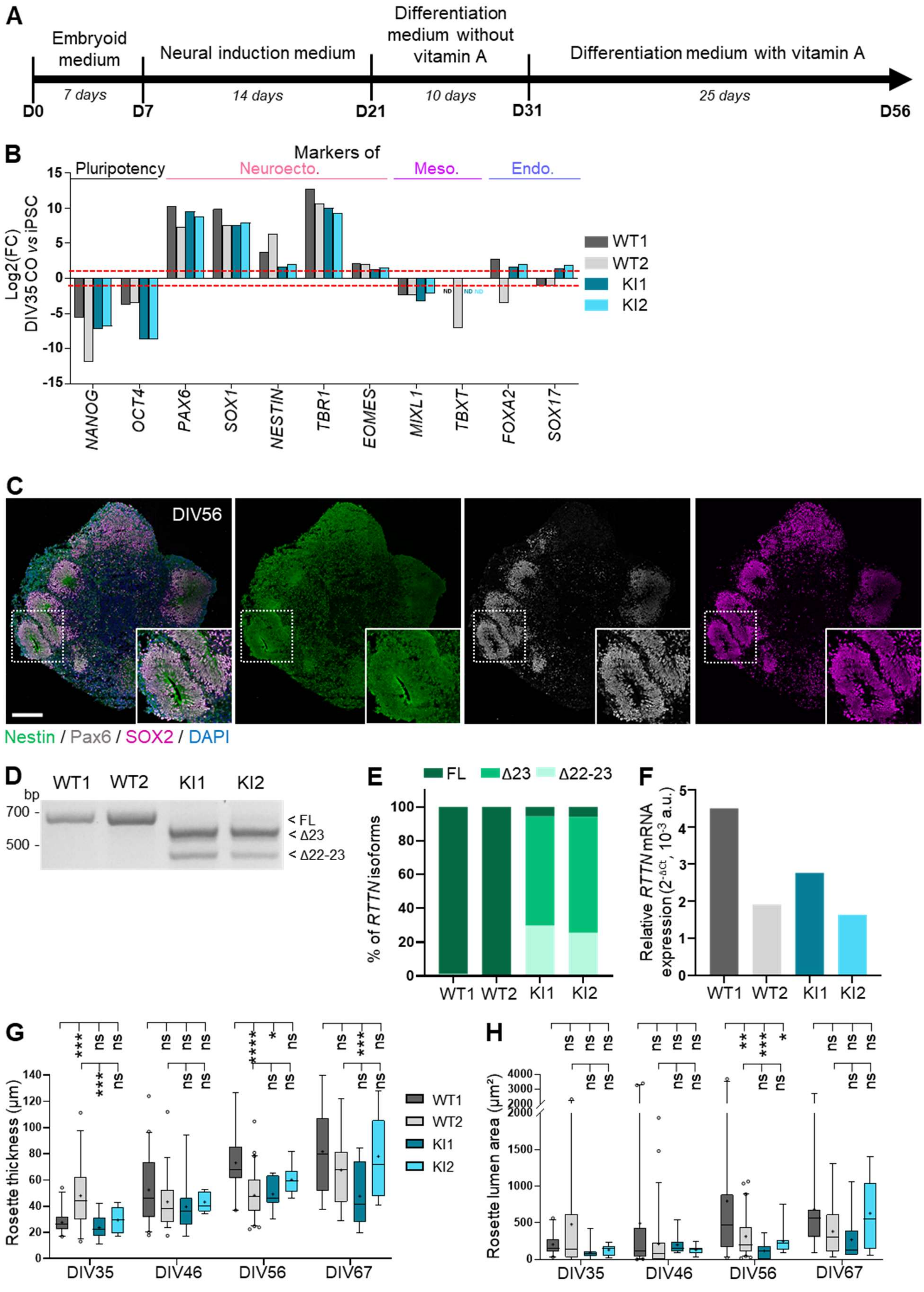
A
Embryoid medium
Neural induction medium
Differentiation medium without vitamin A
Differentiation medium with vitamin A
7 days
14 days
10 days
25 days
D0
D7
D21
D31
D56
B
Markers of
Pluripotency
Neuroecto.
Meso.
Endo.
Log2(FC) DIV35 CO vs iPSC
WT1
WT2
KI1
KI2
NANOG
OCT4
PAX6
SOX1
NESTIN
TBR1
EOMES
MIXL1
TBXT
FOXA2
SOX17
ND
C
DIV56
Nestin / Pax6 / SOX2 / DAPI
D
WT1
WT2
KI1
KI2
bp
700
500
< FL
< Δ23
< Δ22-23
E
FL
Δ23
Δ22-23
% of RTTN isoforms
F
Relative RTTN mRNA expression (2^-ΔCt, 10^-3 a.u.)
G
Rosette thickness (µm)
DIV35
DIV46
DIV56
DIV67
H
Rosette lumen area (µm²)

S9 Fig. 3D differentiation of iPSC into cortical organoids. All experiments were performed in wild-type (WT) and *RTTN*-mutated (KI) cortical organoids (CO). **(A)** Timeline of iPSC differentiation into cortical organoids. **(B)** RT-qPCR analysis of gene expression of markers of pluripotency, neuroectoderm, mesoderm and endoderm in CO at DIV35 compared to iPSC. iPSC express markers of pluripotency while CO predominantly express markers of neuroectoderm. Graph shows the results of one single experiment. **(C)** Representative confocal images of wild-type CO at DIV56. Nestin (green), Pax6 (grey) and SOX2 (magenta) label the NSC organized into neural rosettes (insets). DAPI stains nuclei. **(D-F)** RT-PCR (D) and RT-qPCR (E) analyses of the splicing events of *RTTN* exon 23, and of *RTTN* relative expression in CO at DIV35. *RPS17* was used as a house-keeping gene. Graphs show one single experiment of the pool of 8 organoids. **(G, H)** Quantification of thickness (G) and lumen area (H) of rosettes such as seen in Fig 6A. Box-and-whisker plots show in the box the median (the mean by the cross) and the 25th-75th percentiles, and in whiskers the 5th to 95th percentile of values from 4 different organoids (n=6 to 20 slices total). ns, non-significant; *p-value<0.05; **p-value<0.01; ***p-value<0.001; ***p-value<0.0001 following two-way ANOVA with Tukey's correction (G, F). Scale bar: 200 µm. a.u., arbitrary units; ND, not detected.
