# Supplementary material for: A Taybi-Linder syndrome-related RTTN variant impedes neural rosette formation in human cortical organoids
Source: PLoS Genet. 2024 Dec 16;20(12):e1011517. doi: 10.1371/journal.pgen.1011517 (PMC11684760; doi:10.1371/journal.pgen.1011517)
Supplement: S2 Table — (PDF) [file pgen.1011517.s012.pdf]

S2 Table. Primer sequences

**RT-PCR primer sequences for *RTTN***		
**Targeted region**	**Primer F**	**Primer R**
Exon 21 – Exon 25	TGTGTGAGTCAAGATGGCAAG	TTGCAGCCTGAACAATGGAATG

**RT-qPCR primer sequences for *RTTN***		
**Targeted isoform(s)**	**Primer F**	**Primer R**
All	CATCAGCATTTGTTCAAAGATCTG	GCAGTGGTAAGTTTCAGCTTTCA
Full-length	CTGTTTCCGTTTTTAGAAGGTAC	
Δ23	ATCGAGAATGGATATGTGGTAC	CGGCTTCAAGGCCAAACAAT[a]
Δ22-23	CCGTGTTATTCAGAGGTACCA	

**PCR primer sequences to sequence gDNA after CRISPR-Cas9 experiment**		
**Gene**	**Primer F**	**Primer R**
RTTN	TCATTTGCAGTCAACGAAGTGAG	AGGACCATGAAAGCCCAAGTTA
FGF14	CATCCTTGGTTCCATCGAGA	CCCTGAGCTCACAAGTAGAG
LRRC16A	ATACAACACCTGGCGTTAGGC	CAGGAGCAACGAAAGGGGATTA
ATP8B1	CACTAGAAGCTATAAACGCACTTC	TTAGTCCAAGTGTCTTCCAGCAG
LINC00299	ATGAGCCCTTTTCCTAGATCTGAC	CTCAGAAAAGGGAGCTAAGAAGC

**RT-qPCR primer sequences to analyze expression of differentiation markers**		
**Gene**	**Primer F**	**Primer R**
RPS17	CATTATCCCCAGCAAAAAGC	AGGCTGAGACCTCAGGAACA
NANOG	AAATACCTCAGCCTCCAGCAG	TGCGTCACACCATTGCTATTC
OCT4	AAACCCACACTGCAGCAGATCA	TCCTCTCGTTGTGCATAGTCG
PAX6	CCAACCAATTCCACAACCCA	GTGAGGGCTGTGTCTGTTCG
SOX1	GCTGACACCAGACTTGGGTTT	CCCCTCGAGCAAAGAAAACG
EOMES	GTGGCAAAGCCGACAATAACA	CCTGTCTCATCCAGTGGGAAC
TBR1	CGTGCAGACGTTCACTTTCC	TGTAATATCCGTGTTCTGGTAGGC
NESTIN	CTCAGCTTTCAGGACCCCAAG	GCAAAGATCCAAGACGCCG
TBXT	GCTTCAAGGAGCTCACCAATG	AGACACGTTCACCTTCAGCA
MIXL1	GCAAGCGCACGTCTTTCAG	CGCAAGTGGATGTCGGGGTA
SOX17	ATGTGTCCCAAAACAGCTTCC	ACACACCCAGGACAACATTTCT
FOXA2	CCCCCTACGCCAACATGAAC	TAGCTGCGCCTGTAGGTCTT

**crRNA and ssODN sequences for *RTTN* editing by CRISPR-Cas9**	
crRNA[b]	3'- TTTCCGTTTTTAGAAGGTAA *AGG* -5'
ssODN[c]	GTCTTCAGTTTGCCTGTTTCCGTTTTTAGAGGgtaaaagatttatttctctgattttcttgaaggacg

[a]Same reverse primer for all three transcripts

[b]PAM sequence in italic

[c]Underlined nucleotides are those modified to introduce the c.2953A>G variant and to alter the PAM
