# Supplementary material for: A Taybi-Linder syndrome-related RTTN variant impedes neural rosette formation in human cortical organoids
Source: PLoS Genet. 2024 Dec 16;20(12):e1011517. doi: 10.1371/journal.pgen.1011517 (PMC11684760; doi:10.1371/journal.pgen.1011517)
Supplement: S3 Table — (PDF) [file pgen.1011517.s013.pdf]

S3 Table. Culture media for 2D neuronal differentiation protocol

Product	Supplier	Catalog number	Initial concentration	Final concentration	Dilution
**N2B27 Medium**					
DMEM/F12	Gibco	21331020	-	50%	1/2
Neurobasal	Gibco	21103049	-	50%	1/2
N2	Gibco	17402048	100X	1X	1/100
B27 without vitamin A	Gibco	12587010	50X	1X	1/50
Penicilin-streptomycin	Gibco	15140122	-	1%	1/100
L-glutamine	Gibco	25030024	200mM	2mM	1/100
**Neural Induction Medium (NIM)**					
N2B27	-	-	-	100%	1
β-mercaptoethanol	Gibco	31350010	50mM	100µM	1/500
FGF2	STEMCELL Technologies	78003.1	1µg/mL	20ng/mL	1/50
LDN-193189	STEMCELL Technologies	72147	1mM	500nM	1/2000
SB-431542	STEMCELL Technologies	72234	10mM	20µM	1/500
**Neural Expansion Medium (NEM)**					
N2B27	-	-	-	100%	1
FGF2	STEMCELL Technologies	78003.1	1µg/mL	20ng/mL	1/50
EGF	STEMCELL Technologies	78006.1	10µg/mL	10ng/mL	1/1000
BDNF	STEMCELL Technologies	78005	10µg/mL	20ng/mL	1/500

FGF2, Fibroblast growth factor 2; EGF, Epidermal growth factor; BDNF, Brain derived neuronal factor
