# Supplementary material for: A Taybi-Linder syndrome-related RTTN variant impedes neural rosette formation in human cortical organoids
Source: PLoS Genet. 2024 Dec 16;20(12):e1011517. doi: 10.1371/journal.pgen.1011517 (PMC11684760; doi:10.1371/journal.pgen.1011517)
Supplement: S4 Table — (PDF) [file pgen.1011517.s014.pdf]

S4 Table. Culture media for cortical organoids

Product	Supplier	Catalog number	Initial concentration	Final concentration	Dilution
**Embryoid Body Medium (EB)**					
mTeSR Plus	STEMCELL Technologies	100-0276	-	-	1
Y-27632	STEMCELL Technologies	72302	10mM	10μM	1/1000
SB-431542	STEMCELL Technologies	72234	10mM	10μM	1/1000
**Neural Induction Media (NIM)**					
DMEM-F12	Gibco	21331020	-	-	1
L-glutamine	Gibco	25030024	200mM	2mM	1/100
N2	Gibco	17402048	100X	1X	1/100
Non Essential Amino Acid	Gibco	11140035	100X	1X	1/100
Penicilin-streptomycin	Gibco	15140122	-	0.1%	1/1000
β-mercaptoethanol	Gibco	31350010	50mM	100μM	1/500
LDN-193189	STEMCELL Technologies	72147	1mM	250nM	1/4000
SB-431542	STEMCELL Technologies	72234	10mM	10μM	1/1000
**Neural Expansion Media (NEM)**					
DMEM-F12	Gibco	21331020	-	50%	1/2
Neurobasal	Gibco	21103049	-	50%	1/2
N2	Gibco	17402048	100X	0.5X	1/200
B27, with vitamin A (DM1)[a]	Gibco	17504044	50X	0.5X	1/100
B27, without vitamin A (DM2)[a]	Gibco	12587010	50X	0.5X	1/100
L-glutamine	Gibco	25030024	200mM	2mM	1/100
Penicilin-streptomycin	Gibco	15140122	-	0.1%	1/1000
Non Essential Amino Acid	Gibco	11140035	100X	1X	1/100
β-mercaptoethanol	Gibco	31350010	50mM	100μM	1/500
Insuline	Sigma Aldrich	I9278	See lot	6.25μg/mL	See lot

[a]DM1 from DIV21 to DIV30; DM2 from DIV31 to DIV67
