# Supplementary material for: A Taybi-Linder syndrome-related RTTN variant impedes neural rosette formation in human cortical organoids
Source: PLoS Genet. 2024 Dec 16;20(12):e1011517. doi: 10.1371/journal.pgen.1011517 (PMC11684760; doi:10.1371/journal.pgen.1011517)
Supplement: S5 Table — (PDF) [file pgen.1011517.s015.pdf]

S5 Table. Antibodies

Primary antibodies				
**Targeted protein**	**Supplier**	**Catalog number**	**Host species**	**Dilution**
Adenylate cyclase 3	Fisher Scientific	15368114	Rabbit	1/500
ARL13B	Proteintech	17711-1 AP	Rabbit	1/500
Cleaved caspase 3	Cell signaling	9661	Rabbit	1/500
Centrin3	Abnova	H00001070-M01	Mouse	1/5,000
DM1 α-tubulin	Sigma Aldrich	T9026	Mouse	1/10,000
GFP	BD Biosciences	632381	Mouse	1/5,000
Ki67	BD Biosciences	550609	Mouse	1/250
MAP2	Abcam	ab5392	Chicken	1/1000
N-cadherin	BD Biosciences	610921	Mouse	1/500
Nestin	Novus/Bio-techne	NB100-1604	Chicken	1/500
p21	Fisher Scientific	R.229.6	Rabbit	1/500
p53	Fisher Scientific	DO-7	Mouse	1/200
Pax6	BD Biosciences	561462	Mouse	1/100
Pericentrin	Abcam	ab220784	Rabbit	1/750
Phospho-vimentin	Clinisciences	D076-3	Mouse	1/500
POC1B	Thermofisher	PA5-24495	Rabbit	1/400
Rotatin	Tang's lab - Taiwan	PMID: 28811500	Rabbit	1/400
Sox2	Invitrogen	14-9811-82	Rat	1/250
TPX2	Bio-techne	NB500-179	Rabbit	1/500
Acetylated α-tubulin	Sigma Aldrich	T6793	Mouse	1/1,000 ; 1/125 (UExM)
Acetylated α-tubulin	Sigma Aldrich	T7451	Mouse	1/400
TUJ1	Thermofisher	15234347	Mouse	1/1,000
ZO-1	BD Biosciences	610966	Mouse	1/300

Secondary antibodies				
**Targeted species**	**Fluorophore**	**Supplier**	**Catalog number**	**Dilution**
Goat anti-chicken	Alexa 488	Invitrogen	A11039	1/1,000
Goat anti-rabbit	Alexa 488+	Invitrogen	A32731	1/1,000
Goat anti-mouse	Alexa 488+	ThermoFisher	A32723	1/1,000
Goat anti-rabbit	Alexa 555+	ThermoFisher	A32732	1/1,000
Goat anti-mouse	Alexa 555+	ThermoFisher	A32727	1/1,000 ; 1/400 (UExM)
Goat anti-rabbit	Alexa 647+	ThermoFisher	A32733	1/1,000
Goat anti-mouse	Alexa 647+	Invitrogen	A32728	1/1,000
Goat anti-rat	Alexa 647+	Thermofisher	A21247	1/1,000
