# Supplementary material for: A Taybi-Linder syndrome-related RTTN variant impedes neural rosette formation in human cortical organoids
Source: PLoS Genet. 2024 Dec 16;20(12):e1011517. doi: 10.1371/journal.pgen.1011517 (PMC11684760; doi:10.1371/journal.pgen.1011517)
Supplement: Attachment — Submitted filename: Guguin et al_Response to Reviewers.docx [file pgen.1011517.s016.docx]

**Reviewer #1**:

**This is well written paper reporting a family with the clinical diagnosis of Taybi-Linder syndrome (TALS), a very rare monogenic disorder previously reported to result from RNU4ATAC mutations. The authors however identified a homozygous RTTN mutation instead that results in generation of 3 different aberrant transcripts as shown by detailled transcript analyses. Other mutations in RTTN have been previously identified in microcephaly, short stature and polymicrogyria with or without seizure” (MSSP, OMIM #614833). The identified allele has been reported in approx. 25% of MSSP families before so seems to represent a founder allele, however the effects of this mutation have not been studied before in detail.**

**The genetic findings in this paper a solid and certainly of interest to the human genetics and paediatrics/pediatric neurology community.**

**RTTN is a centrosomal protein and while previous studies suggest a role in procentriole elongation, cell cycle progression, mitosis and also cilia length regulation, it is not really understood how mutations exactly cause microcephaly. The authors have embarked on very detailled and laborious functional studies to investigate how their identified mutations could cause the CNS phenotype in their family, including use of patient fibroblasts, TP53-RTTN knockout RPE1 cells expressing wildtype and mutant RTTN in a doxycycline inducible manner expression and CRISPR/Cas9-edited pluripotent stem cells (iPSC) that they differentiated into neural stem cells (NSC) or cortical organoids (CO).**

**This functional work likewise is very solid and technically sound and involves a large amount of work, including months-long iPSC differentiation into cortical organoids. It proves pathogenicity of the identified RTTN mutation and I feel it’s a great strength of the paper that the authors have put in so much effort to study the effect of the human disease allele instead of using knockout models.**

**The identified mutation results in reduced levels of RTTN at centrioles and reduced centriole length as well as reduced levels of POC1B, a downstream centriole protein. The author further observe mild cell cycle progression defects upon expression of the mutant RTTN in RTTN Null cells. In contrast to a previous report, the authors don’t observe a cilia length defect in patient fibroblasts while RTTN knockout resulted in marked loss of cilia, likely the mutant RTTN retains enough function. They also see delayed cell cycle re-entry in patient fibroblasts. While no significant hedgehog pathway defect was observed, reduced ciliary ACIII was found in patient fibroblasts.

RTTN deficient iPSC cells showed cell cycle defects and increased cell death and slightly misaligned mitotic spindles. The authors suggest that a higher proportion of KI NSC could be prone to undergo asymmetric divisions and thus to prematurely shift to neurogenic cell divisions, which, as a result, would exhaust the progenitor pool. The authors then generated cortical organoids via neural differentiation of the iPSC (culture time 67 days), revealing delayed rosette formation, less-well organized rosettes, altered NSC polarization and reduced number of mitotic events when using RTTN mutant iPSC (carrying the human mutation).**

**Figure 1:**

**- Resolution in figure 1B appears low in my pdf, if this is a general issue and not down to the compression for the reviewer pdf, I would suggest a higher resolution here.**

> Unfortunately, these images are the only ones we could get from the brain morphology investigation by MR. Despite the multiple attempts, we could not reach the neurologist who followed the patient in Spain, and in the meantime, the clinician we are in touch with lost contact with the patient’s family.

**- I would suggest to add a pedigree of the family as well as a RTTN protein schematic showing the disease causing mutations and marking the associated phenotypic outcomes
- One could also move supp figure 1 A and B into figure 1, I feel it would be helpful for the readers to see this within the main manuscript rather than having to go to the supplement.**
> We thank the reviewer for his/her suggestions to improve the reading of the paper. We included in the revised Figure 1 the pedigree, the RTTN protein structure with disease-causing variants (as listed in Supp. Table 1), and the study of *RTTN* splicing that was initially in supp data. While revising Table 1, we noticed we overlooked a reference that described two additional siblings with *RTTN* variants (PMID:37371259). This was added to the revised Supp. Table 1 and references, and subsequent corrections were made in the manuscript (24 families, 40 patients including the one we describe) (P. 5, lines 79-80; P. 16, line 328).  **Discussion
- Do you have a hypothesis why the patient has 11 pairs of ribs?**

> Congenital absence of the last pair of ribs is a frequent anatomical variation in general population, affecting about 3.4% of individuals (PMID:33711639), with an increased frequency (>5%) in genetic/chromosomal disorders (PMID:2965816). This is an unspecific sign, that also reported in several primary microcephaly/dwarfism syndromes (i.e CPAP-, CEP152-, CDK5RAP2-related MCPH [MIM#613676, MIM#613823, MIM#604804]).

**- “…hypothesize that misregulation of ciliary signaling, deriving from chemical or mechanical cues, could alter NSC organization into neural rosettes in absence of functional Rotatin.” Do you have a hypothesis why microcephaly is then not observed with other bona fide ciliopathies such as BBS?**
> Several studies point out the role of primary cilium disassembly as a regulator of the proliferation rate of neuronal progenitors, which when altered in presence of mutations of *WDR62* (PMID:31197141), *CPAP* (PMID:26929011) or *RRP7A* (PMID:33199730) – to only cite these studies – contribute to the phenotype of microcephaly. More recent findings on RRP7A function suggest that the primary cilium-dependant TGFb pathway controls the onset of cilium disassembly, and thus the neuronal progenitor re-entry in cell cycle (EMBO Cilia meeting 2022, Koln, Germany, poster P-166). Another study showed that, besides its early role during development in brain patterning, Shh pathway is also involved in expanding the pool of basal radial glial cells (bRGs), a type of neuronal progenitors that is more abundant in human brain (than in mouse) and that is responsible for the neocortex growth and folding (PMID:21274567).

Thus, even though dysfunction of primary cilia is likely not sufficient to lead to microcephaly by itself, there are more and more evidence showing that we cannot ruled out its implication (see reviews PMID:29030052, PMID: 31177551).

The end of the discussion has been modified to take the reviewer’s comment into account (P. 18, lines 389).

**Supplementary clinical table:
- I would mention 11 pairs of ribs and consider an extra column displaying typical reported features of Taybi-Linder syndrome (TALS), or MOPD1 (OMIM #210710)**

**- There is a typo in the Corpus callosum line: “midly” instead of “mildly” in 2 instances.**

> Suggestions of the reviewer have been added to the Supplementary Table 1.

**Reviewer #2:**

**The authors dissect the mechanism of a recurrent RTTN variant in a rare autosomal syndrome characterized by microcephaly and other features. The identification of the recurrent variant c.2953A>G in their patient with clinical features of TALS does not have great news value, but does add to a general understanding of the phenotype associated with this RTTN variant. This is valuable given the rarity of this disorder (note: the authors provided a review on genotype/phenotype in all patients with this variant, which is added as supplemental data, and that is a nice addition to the report). The authors also performed functional cell-based assays and neural organoid assays to investigate the molecular mechanisms of disease associated with this variant, which is where the news value for PLOS Genetics readers really lies. The general mechanistic message (mitotic and spindle abnormalities, increased apoptosis, delayed neural rosette formation etc.) and the use of iPSC/organoid modeling is exciting, however, mathematical significance is not always consistent/clear in multiple Figures in this report, and discussion about these flaws are lacking. I feel the authors are somewhat overselling their data. In my opinion, this report certainly has potential, but requires major revision prior to acceptance in PLOS genetics. I do also want to note that this report was easy to read and data is well presented: thank you!

Major:
1. The authors do not comment at all on the differences between statistical significance/insignificance in iPSC/organoid findings between WT1/2/3 and KI1/2…. and iPSC/organoid data is where the news value of this manuscript lies. Some data are only significant when compared to WT1 but not WT2/3, and some data sets are significant only when comparing one KI line to WT controls, but not the other KI line that assesses the same variant. Examples in graphs in Fig 4J, 6C/D/F, 7C/F, S6G, S7H, S8B, S8C, S9G, S9F. There are definitely trends visible in (some of) the data, but inconsistencies in statistical significance in data also suggests that data should be interpreted with caution. Some findings are stronger than others. The authors need to address that in their manuscript results/discussion sections and rebuttal. Please, appropriately soften selected results and conclusions throughout the manuscript.**

> We agree that based on the phenotypic variability seen between clones, we should be more precocious with our conclusions, which we tempered throughout the manuscript where it was appropriate. For that matter, because of clonal inconsistencies, we preferred to not conclude affirmatively regarding ciliary length in iPSC (Fig. S6H) and NSC (Fig. S8C), or centriole length in NSC (Fig. S7H).

That said and to answer the reviewer’s comment on clonal variability, we analysed several WT clones before choosing those which were phenotypically closer to the polyclonal/native cell line. As the reviewer noticed, after studying NSC, we had to drop the WT2 cell line and choose the WT3 clone as it was growing better in 3D. Facing the difficulty of working with clones, we now consider mixing several WT or KI clones for our future projects – as well as reinforcing the robustness of the 3D protocol of CO.

Hence, as suggested by the reviewer, we softened our statements in the Results section (P. 13, lines 260-261 in regards to Fig. 4J; P.14 to P.15 in regards to organoid experiments (Fig. 6, 7 and S9)). In addition to that, we added a comment on clonal variability in the Discussion (P. 17, lines 363-366), and modified the message in the Abstract (P. 3, line 42).

**2. Is polarity initially established in rosettes, but then increasingly lost in KI1/2 clones? That is what it sort of looks like based on Figure 6A, but the quantification was only done in the DIV35 stage. It would be interesting to see quantification of polarity assessments of N-cadherin and ZO-1 in different divisional stages before and after DIV35. Also are cilia ever formed in these rosettes in WT populations and in KI clones? I am asking as you state in the discussion you propose that the problem may be ciliary function rather than formation, but did note that loss of RTTN can result in abnormal cilia formation/delayed disassembly. So, what do you see in organoids, which may better resemble the brain? Is there any cilia formation in WT and KI organoids that project into the centre of these rosettes (comparable to 3D cultures of renal cells that form a ball with a lumen inside wherein cilia project towards the centre)? If cilia form, are there any differences between WT and KI? I also wonder about this as cilia disassembly issues have been reported in Seckel syndrome (PMID: 35883578).**

> We thank the reviewer for all his/her relevant questions. Regarding assessment of polarity, we only did N-cadherin staining at DIV35, DIV46, DIV56 and DIV67. No other time points were studied in the time course of CO development. Notably, we did not explore earlier time points because at DIV31, we change 3D culture medium (addition of vitamin A) to favor differentiation, and rosettes appear only from this stage onwards. As shown in Figure R1, differences of N-cadherin focus number were only seen at DIV35. Therefore, we focused ZO-1 and ARL13B analyses at this same time point. Unfortunately, we cannot perform a new 3D culture in the time frame of the revision process, and we agree with the reviewer that further investigations would be necessary (P. 15, line 300).

**Figure R1.** **Quantification of the number of N-cadherin foci observed in CO.** Box-and-whisker plots display, in the box, the median (middle bar), the mean (cross) and the 25th-75th percentiles, and in whiskers, the 5th to 95th percentile of values from 4 different organoids (n=6 to 20 slices in total). ns, non-significant; *p-value<0.05; **p-value<0.01; ****p-value<0.0001 following two-way ANOVA with Tukey’s correction.

Regarding the formation of primary cilia in cortical organoids, they do form at the apical side of NSC and project into the rosette lumen. To illustrate the ARL13B focus quantification we performed in Fig. 6F, we did high magnitude imaging (Figure R2). As can appreciate the reviewer, it is challenging to assess the number and length of primary cilia in this model. For the number, we cannot determine the number of nuclei/cells involved in the rosette, and for the length, cilia are so intermingled (even in a single slice) that it is difficult to precisely determine their length. Of note, we rather attribute the smaller area covered by cilia to the misorganisation of neural rosette in KI condition than to the decrease number of cilia.

**Figure R2. Representative confocal images of centrosome/cilia in neural rosettes of CO.** DIV35 CO cryosections were labelled with SOX2, a marker of progenitor cells (magenta), PCNT for centrosome (green) and ARL13B for cilia (red). Scale bars, 10 µm.

**3. In the discussion of the manuscript the authors state that the RG isoform with p.Arg985Gly rescues as efficiently as WT RTTN. The RG isoform is definitely associated with a better rescue compared to delta 23, however, images such as Fig 2G/2K suggest it does have some impact on centriole length and spindle organization. Also, there is a slight decrease in number of ciliated cells and there seems to be a trend (albeit insignificant) of lower cilium length of +RG compared to +WT in RPE KO cells in Figures S4B and S4C. As such I don’t think that the rescue with +RG is as efficient as +WT. Also, note that not all in silico tools suggest that the amino acid change p.R985Gly is damaging, e.g. the REVEL score (0.37) is in the uncertain range. REVEL is a relatively new and potent prediction tool that I would say is used by most clinical labs these days. Can the authors please adjust their discussion/conclusions? Can they please add the REVEL score?**

> Indeed, rescue experiments performed in RPE1 cells were not all in favor of an absence of pathogenicity of the missense RG variant. This variant does not rescue as efficiently as the WT the cilium formation (number/length), and more importantly, as the reviewer justly noticed, the mitosis defects. Yet, we can argue that the mitosis defects seen in +RG condition (Fig. 4K) do not seem to “be enough” to disturb cell cycle (Fig. 4I). One can hypothesize that a threshold of 40% of normal bipolar mitosis suffice for the cell cycle to occur properly.

Nevertheless, as suggested by the reviewer, we softened our conclusions regarding RG pathogenicity (P.16, lines 336-338) and included the REVEL score in the Discussion (P.16, line 341).

**4. The discussion broadens to RNU4ATAC-associated microcephaly, but what else is known about microcephaly and centrosomes/primary cilia? It seems like there is more information in the literature. Please expand to a broader discussion of microcephaly in this respect (I found the following report in a quick search, but there should be more interesting literature out there: PMID: 35883578).**

> The link between microcephaly and centrosome function is pretty well established. Indeed, most of genes related to microcephaly encode centrosomal proteins, and the main cellular mechanism that is altered in this context is cell proliferation of neuronal progenitors. This notion is mentioned in the Introduction section (P.6, line 91) with the reference of one of the multiple reviews published on the topic.

What is less documented is the role of primary cilium in microcephaly. Several papers now highlight the importance of the timely disassembly of cilium in neuronal progenitors, that can be regulated by centrosomal proteins (CPAP, WDR62, NDE1), or by proteins involved in proteasome (WDR4) for example: if cilium disassembly is delayed, cell proliferation rate is decreased and so is the pool of progenitors. References to these studies have been added to the manuscript (P. 11, line 211; P. 19, line 392). As mentioned to Reviewer #1, another study showed that Shh pathway, known for its role in brain patterning during early development, is also involved at later stages in the expansion of second line progenitors (intermediate progenitors and basal radial glial cells (bRG)) (PMID:21274567). bRG are more abundant in human brain (than in mouse) and are responsible for the neocortex growth and folding.

Hence, there are more and more evidence showing that dysfunction of primary cilium may be involved – in addition to other defects – to microcephaly. The end of the discussion has been modified to that sense (P. 18, lines 389).

**5. Please include references to specific Figures in your Discussion section of the manuscript when discussing your findings.**

> References to the figures have been added to the revised discussion where it suited.
 **Minor:
1. Please refrain from using the word ‘mutation’. The use of ‘variant’ at least in the context of human genetics is more appropriate. Please, replace the word mutation for variant in the title, abstract and throughout the remainder of the manuscript.**

*>* Indeed, we agree ‘variant’ is more suitable than ‘mutation’ when it is related to patients. We replace the word ‘mutation’ wherever it was appropriate.

**2. Abstract: p.3, line 30: The Taybi-Linder syndrome (TALS) is a rare genetic disorder >>> Taybi-Linder syndrome (TALS) is a rare autosomal recessive disorder…
3. Author summary (p.4, line 55). “…and carries a homozygous mutation, the only recurrent one, in the centrosomal gene RTTN” > “****and is homozygous for a recurrent, pathogenic variant in the RTTN gene that encodes a centrosomal protein.”**

**Note: please refrain from using the word “carry” when it does not relate to carrier testing (i.e. when parents are heterozygous for the variant). There are multiple instances where ‘carry’ is used erroneously. Please correct throughout the manuscript.**

> Thank you for these comments and for explaining the subtlety in the use of “carry”.

**4. Introduction: p.5, line 76: please, add variant in HGVS nomenclature (https://hgvs-nomenclature.org/stable/) and include RefSeq (i.e. NM_xxxx (RTTN):c. and p.). I realize that you did add this information in the Supplementary Table (thank you!), but it is important to also have this information in the main manuscript so scientists do not have to dig deep to find it. Please also specify what type of variant this is here.**

> The information of HGVS nomenclature and type of variant are now added in the introduction. Regarding the splicing isoforms (Δ23 and Δ22-23), we added the protein outcomes, based on RNA sequencing, in the revised Figure 1F, and in the text, page 9, lines 168-169.

**5. You use the word link/linked frequently. Where possible, please, explain. For example, p.6, line 88: “Alterations of all these processes have been linked to microcephaly.” >>>** **Alterations of all these processes are thought to contribute to the development of microcephaly.**

> We explained in the Author summary what we meant with the term “link” between minor splicing and centrosome/primary cilium (P. 4, lines 52-53). We also made the modification suggested by the reviewer on P. 6, line 90.

**6. Results. P7, line 100: Please define what sample you received.**

> We received blood sample from which we extracted DNA. We added this information in the text.

**7. Results. P7, line 104: I am not a clinician, but think it is may be more appropriate to provide percentiles for weight/height. What does “-DS” mean? Standard deviations? There are more growth parameters further down in this paragraph. Please also address those.**

> Our mistake about “-DS”, we meant “-SD” of course. Also, regarding percentiles, they are less appropriate when the measures are extreme like it is for the patient. Nevertheless, we added the percentile information to the text.

**8. Results. P7, line 122. Please add:** **This variant was reported in the ClinVar database as pathogenic (Variation ID: 977819).**
> We added the ClinVar information in the text, where it was suggested.

**9. Figure S7E: please add when cells were harvested in the legend.**

> The information (DIV25) is now added in the legend of Supplementary Fig. 7.

**10. Figure S1C: What is the arch with 4? Is that a transcript not described? Could there be more transcripts that the three investigated (not visible with RT-PCR due to quantity or resolution)? I have no experience with RNAseq and am not that familial with these plots. Please, explain.**

> Indeed, the arch with number 4 (4 reads over 55 total) underlines the use of a cryptic donor splice site for exon 23 that spans in intron 23 (~150bp from the 3’ end of exon 23). We did not detect this longer isoform with additional 150bp by RT-PCR (revised Fig. 1E) when we amplified the cDNA fragment exons 21-25. Nevertheless, we did clone the RT-PCR fragments and could detect by sequencing other variants with the use of variable cryptic donor/acceptor sites.

**11. You found slight different abnormalities in fibroblasts, iPSC, NSC. Are there examples of other studies where cellular defects of fibroblasts, iPSC and organoids are slightly different? If you cannot compare to cortical organoids, are there other types of organoids with slight differences compared to iPSC and other cell types in literature? I am not criticizing data here, but just wondered if others have reported discrepancies in other studies (unrelated to RTTN).**

> One example of phenotypic discrepancy between cell types that we can highlight here is the study of Wang *et al*. *JCI* 2021 (PMID: **33630762). They studied *BBS1* and *BBS10* mutations in patients’ fibroblasts and iPSC-derived neurons. They did not observe alterations of ciliogenesis in fibroblasts; however, they did notice longer cilia in *BBS1* or *BBS10* mutated neurons.**

**12. Page 9, line 161: “In patient cells, we observed a reduced quantity of Rotatin…” > “In patient cells, we observed a modest reduction in quantity of Rotatin…”
13. Page 13, line 267: you claim that you see the same proportions of all three isoforms, but the proportion of RG seems lower to me in S9E.**
> We attenuated our statements regarding the expression of RTTN variants where it was suggested.

**Textual:
1. Abstract: p3, line 32: “It is caused by mutations in RNUATAC whose transcript, the small nuclear RNA U4atac, is involved in the excision of ~850 minor introns” > “It is caused by pathogenic variants in the RNUATAC. Its transcript, the small nuclear U4atac, is involved in the excision of ~850 introns”.
2. Abstract: p.3, line34: “…but no mutation in RNU4ATAC, instead the RTN c.2953A>G variant at the homozygous state.” This sentence seems incomplete. Suggest to change to “…****but no pathogenic variant was found in RNU4ATAC, instead a homozygous RTTN c.2953A>G variant was detected by whole-exome sequencing”.
3. Introduction: p.5, line 69: Since the identification in 2011 of RNU4ATAC, … >>> since the identification of RNU4ATAC in 2011, …
4. Introduction: p5, line 74: Here we report on a particular case of a patient with TALS traits but no variant in RNU4ATAC >** **Here we report on a patient with clinical features of TALS but no variant in RNU4ATAC.
5. Introduction: p.5, line 75: carries > has
6. Introduction: p.78… 37 patient with bi-allelic pathogenic variants in RTTN have been reported >>> 37 patients with bi-allelic pathogenic variants in RTTN have been reported (# of these patients from # families had the c.2953A>G variant).
7. Introduction: p.5, line 80: slopping forehead > sloping forehead
8. Introduction: p.5, line 82: adult-hood > adulthood
9. Results: p.7, line 99: TALS-like traits > TALS-like features
10. Results: p.7, line109: “stature weight growth retardation”. I am not sure if such a word exists. Do you mean growth retardation with respect to both weight and height?**

**11. Results: p.7, line 115: microenencephaly. Did you mean microcephaly?
12. Results: p.7, line 120: Then, a whole exome sequencing was done > Then, whole exome sequencing was done
13. Results: p.7, line 121: add RefSeq (NM_xxx) for RTTN
14. Results: p8, line 125: “a boy and a girl, also homozygous…” > “a boy and a girl, are also homozygous…” I am not sure what ‘although not described as such’ refers to in this sentence. Do you mean the authors speak of MOPD1, but not TALS? You did not really explain it clearly, but is TALS a comprehensive term for both MOPDI and MOPDIII?**

**15. Results: p.8, line 133: present quite heterogeneous > present with quite heterogeneous
16. Results: p8, line 137: we undertook to investigate c.2953A>G further through the study of various cellular models, which will also give us insights for the Taybi-Linder syndrome >>>** **we investigated c.2953A>G further through the study of various cellular models, which provided us with insights in the molecular mechanisms of disease associated with Taybi-Linder syndrome.
17. Page 12, line 235: please add at what stage NSC cells were harvested.
18. S9H in the lower graph is mislabeled as “F” in the Figure S9. The legend does correctly refer to H.**

> We thank the reviewer for all these edits that improve the quality of our manuscript.

Regarding point 14, we meant that the boy and the girl were not formally diagnosed with TALS/MOPD1, even though they presented all the features and facial dysmorphy of it. We changed the sentence to make this point clearer. As for MOPDI and MOPDIII, the designations MOPDI/III came from an old radiographic classification, based on atypical pelvic and spinal appearance (PMID: 7201239), nearly 30 years before the discovery of the *RNU4ATAC* variants in 2011. MOPD I and III were suggested to be a same entity as early as 1989 (PMID:2764032), so there's no longer any need to mention “MOPD III”.
