# Supplementary material for: A Taybi-Linder syndrome-related RTTN variant impedes neural rosette formation in human cortical organoids
Source: PLoS Genet. 2024 Dec 16;20(12):e1011517. doi: 10.1371/journal.pgen.1011517 (PMC11684760; doi:10.1371/journal.pgen.1011517)
Supplement: Attachment — Submitted filename: Guguin et al_Response to Reviewers_R2.docx [file pgen.1011517.s017.docx]

**Reviewer's Responses to Questions**

**Reviewer #1:**

**I feel overall the points raised have been addressed sufficiently.**

**Both reviewers raised the point to broaden the discussion of potential microcephaly-cilia-relationships. The authors themselves now state in the discussion page 19/20 that microcephaly is not a clinical features of classic ciliopathies (I agree), my only remaining minor point would be to maybe very briefly discuss here the possibility that ciliary defects or delayed deciliation observed in conjunction with reduced NPC proliferation and subsequent microcephaly may be a secondary effect of cell cycle progression issues, causing both delayed deciliation and reduced proliferation, rather than primarily delayed deciliation causing cell cycle progress issues (this is kind of the hen and the egg question...).**

**If deciliation/cilia length issues are secondary in microcephaly disorders, this could explain the lack of microcephaly phenotypes in primary ciliary diseases.**

I agree with the reviewers that, in the case of RTTN, alterations for mitotic spindle pole number are prevalent and are certainly the main cause of microcephaly in patients. Nevertheless, I believe that other alterations contribute to this phenotype: we showed that even if progenitor cells manage to correctly duplicate their centrosome and undergo cell division, the mitotic axis orientation may be impaired, thus resulting, in addition to defective cell division, to premature neuronal differentiation. To my point of view, ciliary defects (formation, disassembly, signaling) can also participate – of course to a lesser extent – to microcephaly.

Saying that, I also agree that primary cilium defects can be secondary to mitotic defects (case of KIF14, PMID: 30388224). Hence, I opened the discussion to include this point of view that overlooked in the first revised manuscript.
